# Elevation of enterococcus-specific antibodies associated with bacterial translocation is predictive of survival rate in chronic liver disease

**DOI:** 10.3389/fmed.2022.982128

**Published:** 2022-08-11

**Authors:** Motoh Iwasa, Akiko Eguchi, Yasuyuki Tamai, Ryuta Shigefuku, Ryo Nakagawa, Hiroshi Hasegawa, Jumpei Kondo, Masayuki Morikawa, Eiji Miyoshi, Hayato Nakagawa

**Affiliations:** ^1^Department of Gastroenterology and Hepatology, Mie University Graduate School of Medicine, Tsu, Japan; ^2^Omiya City Clinic, Omiya, Japan; ^3^Department of Molecular Biochemistry and Clinical Investigation, Osaka University Graduate School of Medicine, Osaka, Japan; ^4^Mie Prefectural Mental Care Center, Tsu, Japan

**Keywords:** chronic liver disease, liver cirrhosis, *Enterococcus faecalis*, bacterial translocation, mortality, rifaximin

## Abstract

**Introduction/purpose:**

The gut-liver axis contributes to disease progression, a rise in infection rate, organ failure and a poor overall outcome in chronic liver diseases (CLD). Monitoring of the gut-liver axis is critical in understanding disease status, but biomarkers have not been elucidated. The aim of this study is to determine the level of serum antibodies against *Enterococcus (E.) faecalis* in evaluating patients with CLD, including those treated with rifaximin (a minimally absorbed antibiotic), and in patients with alcohol-associated liver disease (ALD).

**Materials and methods:**

We enrolled 109 CLD patients (cohort 1), 30 hepatic encephalopathy patients treated with rifaximin (cohort 2), 53 inpatients with ALD undergoing alcohol cessation (cohort 3) and 33 healthy subjects. To assess the consequences of *E. faecalis* translocation, we developed an assay for the detection of a serum antibody against *E. faecalis* capsular polysaccharide (*E.*CPS).

**Results:**

Serum *E.*CPS antibody titer was elevated only in those patients with advanced CLD and ALD. The *E.*CPS antibody titer was an independent prognostic factor (*p* < 0.05), while Mac-2 binding protein and albumin-bilirubin score were not independent predictors of survival. The improvement of predictive model in integrated factors was significant [continuous net reclassification index (value 0.699, *p* < 0.05) and integrated discrimination improvement (value 0.164, *p* = 0.051)]. Furthermore, rifaximin treatment led to a decrease of serum *E.*CPS antibody titer resulting in a significantly longer overall rate of survival.

**Conclusion:**

The *E.*CPS antibody titer appears to be a strong predictor of survival in CLD patients. Serum *E.*CPS levels decrease in CLD patients receiving rifaximin, and may be associated with an overall improvement in rate of survival.

## Introduction

Fecal dysbiosis, small intestinal bacterial overgrowth, intestinal epithelial barrier dysfunction and increased permeability have been described as key events in patients with advanced chronic liver diseases (CLD) (1). Gut dysbiosis may contribute to translocation of pathological pathogen-associated molecular patterns including capsular polysaccharide (CPS), but a dysfunctional/leaky gut barrier plays a greater role, which has been shown to contribute particularly to bacterial translocation (BT) in alcohol-related cirrhosis (2, 3). This gut-liver axis has been identified as a contributing factor to disease progression, increase of infection rates, development of complications, organ failure and generally poor outcomes related to CLD (3), although complex mechanisms remain poorly understood.

The most common tools used to predict the overall outcome of CLD patients include the following: assessing severity of portal hypertension, scoring systems such as the model of end-stage liver disease (MELD) and Child-Pugh score, and blood biomarkers related to complications and/or survival rate (4). Recently, several biomarkers have been proposed based on the importance of BT in the clinical course of CLD, which include bacterial DNA, soluble CD14, endotoxin and lipopolysaccharide-binding protein (LBP) (5) and the number of potential biomarkers is increasing based on discovery of many mechanisms (6, 7). However, there are no unique universally recognized biomarkers that are used in clinical practice as a surrogate for the presence of BT.

The exotoxin-secreting gut bacterium *Enterococcus (E.) faecalis* is a critical contributor to the progression of alcoholic hepatitis (8) and pancreatic diseases (9), and it is also recognized as a key promoter of liver carcinogenesis (10). This suggests that the monitoring of *E. faecalis* may be useful in the clinical determination of overall disease status. Indeed, we previously demonstrated an increase in serum antibody titer against *E. faecalis* CPS (*E.*CPS) in patients with chronic pancreatitis and pancreatic cancer (9), as well as cirrhotic rats (11). In addition, we found that rifaximin, a minimally absorbed antibiotic, attenuated not only hyperammonemia, but also serum *E.*CPS antibody titer and intestinal inflammation (12).

In this study, we investigate the utility of monitoring serum antibody levels against *E.*CPS as a disease survival predictor and compare it with LBP (BT marker), Mac-2, as a cell-associated macrophage antigen, binding protein (Mac-2 bp; hepatocyte damage or hepatic condition marker) (13), Aleuria aurantia lectin-haptoglobin (AAL-Hp; hepatocyte damage or hepatic condition marker) (14) and albumin-bilirubin (ALBI; hepatic functional reserve marker) score in patients with CLD. We also investigate the association between the changes in *E.*CPS antibody levels after rifaximin treatment and overall rates of mortality in patients with hepatic encephalopathy. In addition, we evaluated the changes in *E.*CPS antibody titer in patients with alcohol-associated liver disease (ALD) during a period of alcohol cessation.

## Materials and methods

### Patients and methods

The study protocol was approved by the ethics committee of Mie and Osaka Universities, Mie Prefectural Mental Care Center, and Omiya City Clinic. This study was performed retrospectively on stored samples, and subjects were allowed to opt out of their data being used. Written informed consent was obtained from all subjects at the time of blood sampling. A total of 109 patients were recruited by their stage of CLD for this study (cohort 1). Patients positive for hepatitis B surface antigen were diagnosed with hepatitis B virus (HBV) infection, whereas those positive for hepatitis C virus (HCV) RNA were diagnosed with HCV infection. ALD was defined as the presence of alcohol consumption >60 g/day. The diagnosis of liver cirrhosis and hepatocellular carcinoma (HCC) were based on clinical history, serologic testing, and radiologic imaging and HCC patients were grouped by the Barcelona Clinic Liver Cancer (BCLC) staging classification (15). Patients who had other malignancies within the past 3 years, severe hepatic failure (MELD score ≥30), uncontrollable infection, heart failure greater than the New York Heart Association-defined category of class II, human immunodeficiency virus infection, pregnancy or psychiatric problems were deemed to be unsuitable for clinical study. All treatments for HCC were performed following the Japanese practical guidelines for HCC as possible. As a general rule, the follow-up examinations included routine physical examinations and biochemical tests (1–3 monthly) and diagnostic imaging studies including ultrasonography.

Thirty hepatic encephalopathy patients treated with rifaximin were enrolled to investigate the association between the changes in *E.*CPS antibody levels and mortality (cohort 2). A separate 53 inpatients with ALD who had been treated with alcohol cessation intervention were enrolled as cohort 3. In cohort 3, alcohol abstinence was confirmed by the percent carbohydrate deficient transferrin (%CDT) using the N Latex CDT direct immuno-nephelometric assay (Siemens Healthcare Diagnostics, Kawasaki, Japan) (16). Thirty-three subjects (25 male and 8 female) whose liver function tests were within normal levels were also enrolled as healthy controls.

Clinical records including alanine aminotransferase (ALT), aspartate aminotransferase (AST), albumin (ALB), glutamyl-transferase (GGT), total bilirubin (T-bil), sodium (Na), blood urea nitrogen (BUN) and creatinine (Cr) were retrospectively evaluated. Blood samples were kept at −80 degrees until *E.*CPS antibody, LBP, Mac-2 bp and AAL-Hp measurements could be performed. The presence of infection with *E. faecalis* was evaluated using a CPS-specific ELISA (17). A lectin-antibody ELISA was used to determine serum AAL-Hp levels, as described previously (18). The serum LBP and Mac-2 bp levels were determined using an ELISA kits (R&D systems, Minneapolis, MN, United States and Immuno-Biological Laboratory, Gunma, Japan, respectively) (13) according to manufacturer’s instruction. The body mass index (BMI), Child-Pugh score, ALBI score and fibrosis index based on 4 factors (FIB-4) were calculated.

### Statistical analyses

Continuous variables are presented as mean ± standard deviation or median (25th and 75th percentiles), and categorical variables are shown as numbers of patients. Data were analyzed using the Mann-Whitney *U*-test in two groups and one-way analysis of variance for comparison of continuous variables. The categorical data were compared using the Chi-squared test. The relationship between the serum *E.*CPS antibody titer and clinical data were examined using Spearman’s rank correlation coefficient. Receiver operator characteristic (ROC) curves and the corresponding area under the curve (AUC) were used to obtain cut-offs for the outcomes. The Youden index was applied to calculate the optimal cut-off point. We also conducted time-dependent ROC analysis for the prediction of CLD patient survival or death based on duration after CLD diagnosis. Overall survival (OS) was measured using the Kaplan-Meier method and compared using the log-rank test. Associations between predictor variables and OS were determined by the hazard ratio (HR) and 95% confidence interval (CI) calculated using Cox proportional hazards regression. The improvements on the basic model were assessed by the continuous net reclassification index (NRI) and integrated discrimination improvement (IDI) of which values above 0 were regarded as significant. We further conducted time-dependent concordance index (C-index) analysis for the prediction of CLD patient survival, or death, based on the duration of time post CLD diagnosis. For cohort 2 in post-rifaximin, we combined patients who had *E.*CPS at 3 and 6 months available to do the analysis and defined rifaximin effective patients with below 0.018 *E.*CPS antibody levels at 3 or 6 months or at both 3 and 6 months in the same patients. The statistical analyses were performed using the JMP software program (SAS Institute, Cary, NC, United States) for univariate and multivariate logistic regression analyses. Differences were considered to be significant at *p* < 0.05.

## Results

### Patient demographics in patients with chronic liver diseases

Clinical features of the 109 CLD patients (cohort 1) are shown in Table 1. Cohort 1 study patients were admitted to our investigation based on a variety of causative agents: 27 HBV, 63 HCV, and 19 patients with ALD. Patients infected with HBV or HCV were under infection control, with sustained virological response monitoring, by direct-acting antiviral treatment against HCV, or treatment with nucleos(t)ide analogs against HBV in the clinical course of each patient. 50 liver cirrhotic patients composed of 32 patients with Child-Pugh class A and 17 patients with Child-Pugh class B and C. We identified 18 patients (15 patient with Child-Pugh class A and 3 with Child-Pugh class B and C) who presented with HCC in addition to underlying liver cirrhosis.

**TABLE 1:** Demographic and clinical characteristics of subjects.

Parameter	CH (*N* = 59)	LC (*N* = 50)	*P*-values
Age (year)	69.0 (58.0, 74.0)	66.5 (58.8, 74.3)	0.9167
Gender (M/F)	31/28	39/11	0.0057**
Etiology (HBV/HCV/alcohol)	18/41/0	9/22/19	<0.0001****
Child-Pugh (A/B-C)		32/17	
Child-Pugh (A/B-C) in 18 HCC		15/3	
HCC (-/ +)	59/0	32/18	<0.0001****
BCLC (A/B/C/D) in 18 HCC		17/0/1/0	
BMI (kg/m^2^)	22.7 (19.5, 24.5)	23.8 (20.3, 25.4)	0.0144
AST (U/L)	24.0 (20.0, 31.0)	31.0 (24.8, 38.8)	0.0812
ALT (U/L)	18.0 (13.0, 25.0)	19.0 (14.0, 27.0)	0.6032
ALB (g/dL)	4.40 (4.20, 4.60)	3.90 (3.40, 4.30)	<0.0001****
T-bil (mg/dL)	0.80 (0.60, 1.00)	1.10 (0.90, 1.50)	<0.0001****
GGT (U/L)	19.0 (15.0, 32.0)	41.5 (22.8, 83.8)	<0.0001****
FIB-4 index	2.25 (1.49, 3.28)	4.78 (2.60, 7.04)	<0.0001****
Na (mEq/L)	141.0 (140.0, 142.0)	140.0 (138.0, 142.0)	0.0263*
BUN (mg/dL)	14.5 (12.6, 16.6)	15.8 (12.9, 18.7)	0.1478
Cr (mg/dL)	0.73 (0.59, 0.91)	0.86 (0.71, 1.08)	<0.0001****

HBV, hepatitis B virus; HCV, hepatitis C virus; HCC, hepatocellular carcinoma; BCLC, barcelona clinic liver cancer; BMI, body mass index; ALB, albumin; ALT, alanine aminotransferase; AST, aspartate aminotransferase; Na, sodium; BUN, blood urea nitrogen; Cr, creatinine; T-bil, total bilirubin; GGT, glutamyl-transferase; FIB-4, Fibrosis-4.

Statistics include number (%) or median (25th and 75th percentiles). ****P < 0.0001, **P < 0.01, *P < 0.05.

### Correlation of lipopolysaccharide-binding protein, *Enterococcus faecalis* capsular polysaccharide antibody, Mac-2 bp and aleuria aurantia lectin-haptoglobin to clinical parameters in all patients

The correlations between LBP, *E.*CPS antibody, Mac-2 bp or AAL-Hp and clinical parameters in CLD patients are shown in Table 2. The serum LBP levels were significantly correlated with serum AST, ALT, GGT, and Cr levels. Serum *E.*CPS antibody titer was significantly correlated with AST, hepatic function (ALB and T-bil) and fibrosis markers (FIB-4) and Cr values. Mac-2 bp was significantly correlated with transaminases, hepatic function, fibrosis markers, and Cr values.

**TABLE 2:** Correlation of LBP, *E.*CPS, Mac-2 bp and AAL-Hp to clinical parameters in all patients.

	AST	ALT	GGT	ALB	T-bil	BUN	Cr	Na	FIB-4
LBP	**0.20** ***P* = 0.033**	**0.21** ***P* = 0.024**	**0.46** ***P* < 0.0001**	−0.17 *P* = 0.071	0.04 *P* = 0.676	0.13 *P* = 0.165	**0.29** ***P* = 0.0025**	−0.19 *P* = 0.072	−0.00 *P* = 0.951
*E.*CPS	**0.41** ***P* < 0.0001**	0.09 *P* = 0.38	0.10 *P* = 0.303	−**0.45** ***P* < 0.0001**	**0.24** ***P* = 0.014**	0.05 *P* = 0.628	**0.21** ***P* = 0.029**	−0.15 *P* = 0.169	**0.46** ***P* < 0.0001**
Mac-2 bp	**0.39** ***P* < 0.0001**	**0.22** ***P* = 0.021**	**0.49** ***P* < 0.0001**	−**0.39** ***P* < 0.0001**	**0.22** ***P* = 0.023**	0.15 *P* = 0.124	**0.87** ***P* < 0.0001**	−**0.23** ***P* = 0.026**	**0.34** ***P* = 0.0002**
AAL-Hp	0.05 *P* = 0.62	−0.001 *P* = 0.99	0.18 *P* = 0.064	−0.10 *P* = 0.322	−0.12 *P* = 0.222	0.10 *P* = 0.282	0.07 *P* = 0.443	−0.04 *P* = 0.697	0.07 *P* = 0.485

Upper digit indicates correlation efficient using spearman and lower digit indicates *p*-values.

ALB, albumin; ALT, alanine aminotransferase; AST, aspartate aminotransferase; Na, sodium; BUN, blood urea nitrogen; Cr, creatinine; T-bil, total bilirubin; GGT, glutamyl-transferase; FIB-4, Fibrosis-4; LBP, lipopolysaccharide-binding protein; *E*.CPS, *E*. faecalis capsular polysaccharide; Mac-2 bp, Mac-2 binding protein; AAL-Hp, aleuria aurantia lectin-haptoglobin.

Bold values indicate a significance *P* < 0.05.

### Serum biomarker levels in healthy individuals, chronic hepatitis, and liver cirrhosis groups

**FIGURE 1:**
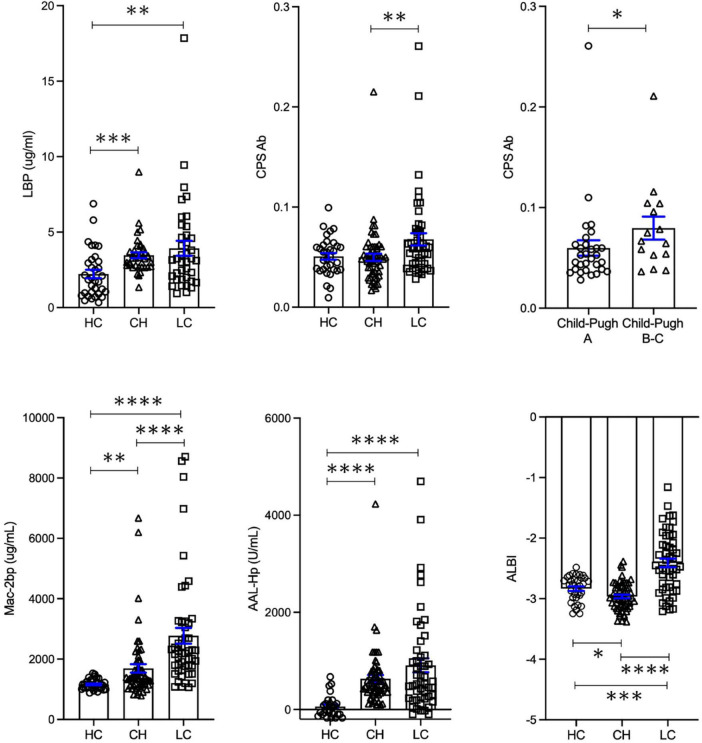
Serum biomarker levels in healthy controls, chronic hepatitis and liver cirrhosis groups. Changes in LBP, *E.*CPS, Mac-2 bp, AAL-Hp and ALBI. HC, healthy controls; CH, chronic hepatitis; LC, liver cirrhosis; LBP, lipopolysaccharide-binding protein; *E.*CPS, *E. faecalis* capsular polysaccharide; Mac-2 bp, Mac-2 binding protein; AAL-Hp, Aleuria aurantia lectin-haptoglobin; ALBI, albumin-bilirubin. *p < 0.05, ^**^p < 0.01, ^***^p < 0.001, ^****^p < 0.0001.

Serum LBP levels were significantly elevated in both chronic hepatitis and liver cirrhosis groups compared to healthy controls (*p* < 0.001, *p* < 0.01, respectively; Figure 1). Serum *E.*CPS antibody titer was significantly elevated in liver cirrhosis patients compared with chronic hepatitis patients (*p* < 0.01). Moreover, *E.*CPS antibody titer was gradually increased in liver cirrhosis progression (Child-Pugh A vs. Child-Pugh B and C, *p* < 0.05; Figure 1). In order to assess degree of hepatocyte damage or overall hepatic condition in this cohort, Mac-2 bp, AAL-Hp and ALBI were also measured. Serum Mac-2 bp levels were significantly elevated in both investigatory groups when compared to healthy controls, as well as significant differences observed between investigatory groups (healthy controls to chronic hepatitis, *p* < 0.001; chronic hepatitis to liver cirrhosis and healthy controls vs. liver cirrhosis, *p* < 0.0001; Figure 1). AAL-Hp levels were significantly elevated in both chronic hepatitis and liver cirrhosis groups when compared to healthy controls (*p* < 0.0001). ALBI score was significantly increased in liver cirrhosis compared to healthy controls or chronic hepatitis (*p* < 0.001, *p* < 0.0001, respectively; Figure 1). Notably, these results were similar in cohort 1 excluding 18 HCC patients (Supplementary Table 1 and Supplementary Figure 1). These results indicate that serum *E.*CPS antibody titer reflects liver cirrhosis progression, while serum LBP, Mac-2 bp, AAL-Hp and ALBI scores can be used to monitor the progression of liver diseases.

### Patient survival

**FIGURE 2:**
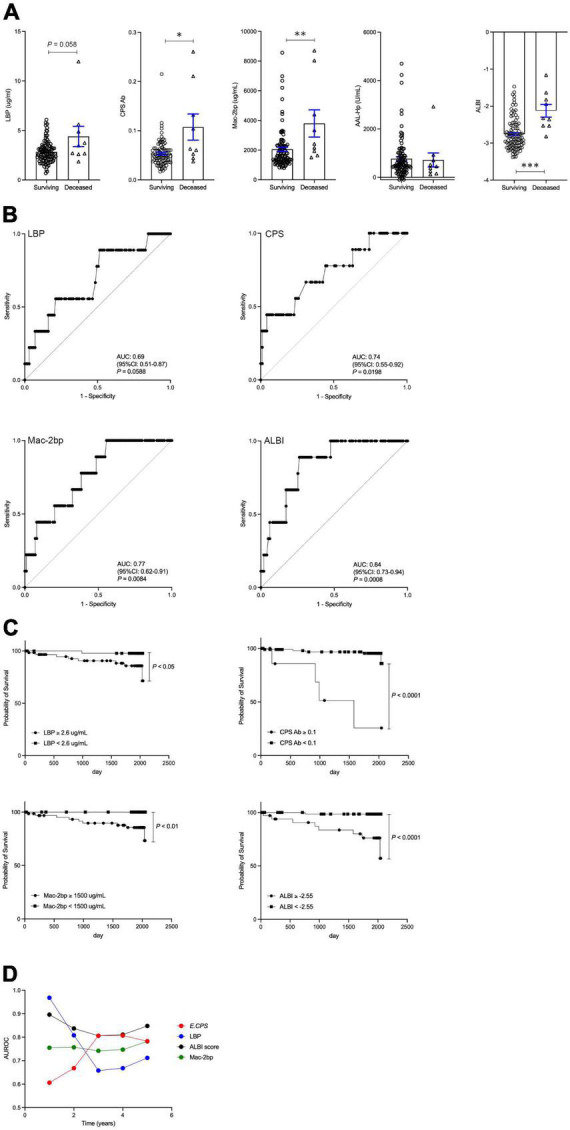
The contributions of LBP, *E.*CPS, Mac-2 bp, AAL-Hp and ALBI score to overall survival. **(A)** Surviving vs. deceased, **(B)** receiver operator characteristic analysis of survival curves, **(C)** survival curves, **(D)** Plots of 1 year AUROC for LBP, *E.*CPS, Mac-2 bp and ALBI score. LBP, lipopolysaccharide-binding protein; *E.*CPS, *E. faecalis* capsular polysaccharide; Mac-2 bp, Mac-2 binding protein; AAL-Hp, Aleuria aurantia lectin-haptoglobin; ALBI, albumin-bilirubin; AUROC, area under the receiver operator characteristic curve; CI, confidence interval. *p < 0.05, ^**^p < 0.01, ^***^p < 0.001.

Nine out of 109 patients (8.3%) died in the average follow-up period of 1652 ± 623 days during our study period. The ultimate cause of death for all patients was considered liver-related. There is no patient received a liver transplant in this study. An increasing trend in serum LBP levels was observed in the deceased group (*p* = 0.058). Serum *E.*CPS antibody titer and Mac-2 bp levels, as well as ALBI score, were significantly increased in the deceased group when compared with the survival group (*p* < 0.05, *p* < 0.01, *p* < 0.001, respectively). In contrast, serum AAL-Hp levels showed no difference between the deceased and survival groups (Figure 2A). ROC analyses concerning predictors of survival yielded AUC values of 0.69 (*p* = 0.0588) for LBP, 0.74 (*p* < 0.05) for *E.*CPS, 0.77 (*p* < 0.01) for Mac-2 bp and 0.84 (*p* < 0.001) for the ALBI score (Figure 2B). From our ROC analysis of survival curves we calculated the cut-off value of LBP at 2.6 μg/mL (sensitivity 88.9% and specificity 48.5%), *E.*CPS at 0.1 (sensitivity 44.4% and specificity 95.9%), Mac-2 bp at 1,500 μg/mL (sensitivity 100% and specificity 44.4%) and the ALBI score at −2.55 (sensitivity 88.9% and specificity 73.7%) (Figure 2B). From Kaplan-Meier survival analysis, we found that patients with low serum LBP levels (<2.6), low serum *E.*CPS antibody titer (<0.1), low serum Mac-2 bp levels (<1500) and a high ALBI score (≥−2.55) showed significantly better OS than patients with high serum LBP levels, high serum Mac-2 bp levels, high *E.*CPS antibody titer and a low ALBI score, respectively (*p* < 0.05, *p* < 0.0001, *p* < 0.01, *p* < 0.0001; Figure 2C). Notably, these results were similar in cohort 1 excluding 18 HCC patients (Supplementary Figure 2). We further conducted time-dependent ROC analysis for the prediction of CLD patient survival, or death, based on the duration of time post CLD diagnosis. AUROC values for LBP were highest in the short-term, but were lowest in the long-term (Figure 2D). In contrast, AUROC values for *E.*CPS were lowest in the short-term and were high in the long-term. AUROC values for Mac-2 bp and ALBI score were constant in the short- and long-term (Figure 2D).

### Investigation of factors affecting overall survival

The contributions of age, gender, ALT, LBP, Mac-2 bp, AAL-Hp, *E.*CPS antibody titer and ALBI score were evaluated by univariate analysis using a Cox proportional hazard model (Table 2). A significant predictor of OS was *E.*CPS antibody titer (*p* < 0.0001), as well as serum LBP (*p* < 0.001), Mac-2 bp levels (*p* < 0.01) and ALBI score (*p* < 0.001). Using multivariate analysis, the *E.*CPS antibody titer and LBP levels were independent prognostic factors (*p* < 0.05, *p* < 0.01, respectively), while serum Mac-2 bp levels and ALBI score were not independent predictors of survival (Table 3). Compared with the basic model, the predictive value of the improved model on favorable prognosis showed a significant improvement when assessed by AUC (basic model 0.850, improved model integrated with LBP plus *E.*CPS 0.869; *p* < 0.001), continuous NRI (value 0.699; *p* < 0.05), and IDI (value 0.164; *p* = 0.051) (Table 4). C-index values for improved model integrated with LBP plus *E.*CPS were constantly high in the short- and long-term (Supplementary Figure 3).

**TABLE 3:** Univariate and multivariate Cox regression analysis for predictors of survival in chronic liver disease patients.

	Univariate			Multivariate		
Variables	HR	95% CI	*P*-value	HR	95% CI	*P*-value
**Gender**						
Male	1.839	0.375–9.011	0.452			
Age	1.030	0.970–1.098	0.349			
ALT	1.021	0.980–1.051	0.209			
LBP	1.973	1.356–1.973	<0.001	1.994	1.278–3.319	<0.01
*E.*CPS	1.187	1.080–1.293	<0.0001	1.182	1.017–1.348	<0.05
**(HR per 0.01 units)**						
Mac-2 bp	1.000	1.000–1.000	<0.01	1.000	1.000–1.000	0.9281
AAL-Hp	0.999	0.999–1.000	0.968			
ALBI score	9.794	2.986–34.28	<0.001	2.641	0.631–13.32	0.1908

ALT, alanine aminotransferase; LBP, lipopolysaccharide-binding protein; *E*.CPS, *E*. faecalis capsular polysaccharide; Mac-2 bp, Mac-2 binding protein; AAL-Hp, aleuria aurantia lectin-haptoglobin; ALBI, albumin-bilirubin; HR, hazard ratio; CI, confidence interval.

**TABLE 4:** Comparison of basic models and models adding factors for predicting favorable prognosis and excellent prognosis.

Variables	C-index	*P*-value	Continuous NRI (95% CI)	*P*-value	IDI (95% CI)	*P*-value
ALBI + Mac-2 bp	0.850	0.001	Reference		Reference	
ALBI + Mac-2 bp + *E.*CPS	0.857	<0.001	0.353 (−0.320, −1.026)	0.304	0.099 (−0.313, −0.229)	0.136
ALBI + Mac-2 bp + LBP	0.853	<0.001	0.020 (−0.623, −0.663)	0.951	0.055 (−0.064, −0.174)	0.368
ALBI + Mac-2 bp + *E.*CPS + LBP	0.869	<0.001	0.699 (0.0298, −1.368)	0.041	0.164 (−0.001, −0.328)	0.051

ALBI, albumin-bilirubin; Mac-2 bp, Mac-2 binding protein; *E*.CPS, *E*. faecalis capsular polysaccharide; LBP, lipopolysaccharide-binding protein; C-index, concordance index; CI, confidence interval; NRI, net reclassification improvement; IDI, integrated discriminating improvement.

### The changes in *Enterococcus faecalis* capsular polysaccharide antibody titer after rifaximin treatment and survival

**FIGURE 3:**
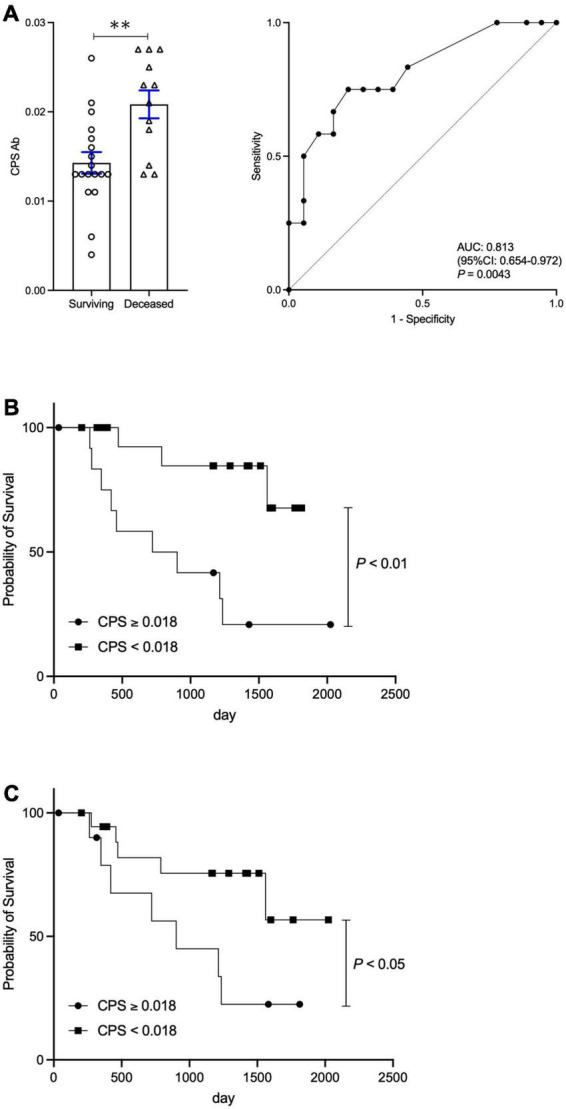
Change in *E.*CPS antibody titer after rifaximin treatment and survival. **(A)** Surviving vs. deceased and Receiver operator characteristic analysis of survival curves, **(B)** survival curves of low and high *E.*CPS antibody titer at baseline, **(C)** survival curves of low and high *E.*CPS antibody titer at both 3 and 6 months post-rifaximin treatment. *E.*CPS, *E. faecalis* capsular polysaccharide; AUC, area under the curve; CI, confidence interval. ^**^p < 0.01.

Cohort 2 consisted of 30 hepatic encephalopathy patients treated with rifaximin, a group previously investigated (12). Briefly, we reported that rifaximin treatment attenuated not only hyperammonemia, but also *E.*CPS antibody titer. Twelve out of 30 patients (40%) died in the average follow-up period of 977.5 ± 578.5 days during our study period. *E.*CPS antibody titer was significantly elevated in the deceased group (*p* < 0.01; Figure 3A). ROC analyses concerning predictors of survival yielded AUC values of 0.813 (*p* < 0.01) for *E.*CPS (Figure 3A). In the present study, we calculated the cut-off value of *E.*CPS antibody titer at 0.018 (sensitivity 75.0% and specificity 77.8%) from our ROC analysis of survival curves. Patients with low *E.*CPS antibody titer (<0.018) showed significantly better OS than patients with high *E.*CPS antibody titer (*p* < 0.01; Figure 3B). Moreover, 19 out of 30 patients with low *E.*CPS antibody titer (<0.018), at 3 or 6 months post-rifaximin treatment or at both 3 and 6 months post-rifaximin treatment in the same patients, showed significantly better OS than patients with high *E.*CPS antibody titer (≥0.018) at 3 or 6 months (*p* < 0.05; Figure 3C). These results show that serum *E.*CPS antibody titer can be useful for predicting rate of survival.

### Biomarker changes in patients with alcohol-associated liver disease during alcohol cessation

**FIGURE 4:**
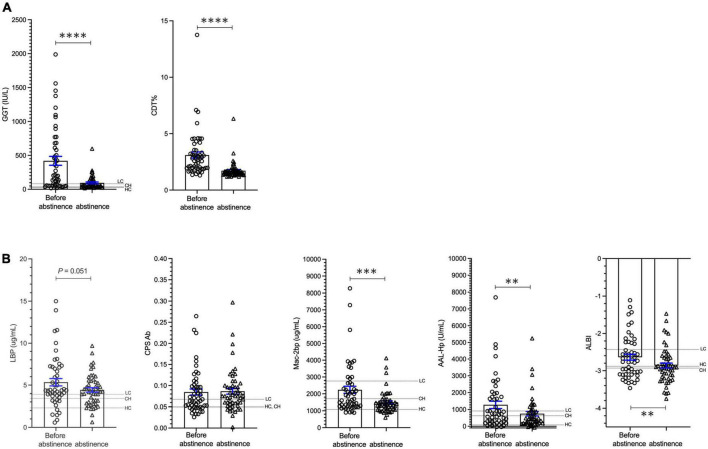
Changes in biomarkers in patients with ALD during alcohol cessation. **(A)** Changes in GGT and CDT. **(B)** Changes in LBP, *E.*CPS, Mac-2 bp, AAL-Hp and ALBI. GGT, glutamyl-transferase; CDT, carbohydrate deficient transferrin; LBP, lipopolysaccharide-binding protein; *E.*CPS, *E. faecalis* capsular polysaccharide; Mac-2 bp, Mac-2 binding protein; AAL-Hp, aleuria aurantia lectin-haptoglobin; ALBI, albumin-bilirubin; HC, healthy control; CH, chronic hepatitis; LC, liver cirrhosis. The mean values in HC, CH and LC subjects are indicated. ^**^p < 0.01, ^***^p < 0.001, ^****^p < 0.0001.

In cohort 3, the mean patient age was 56.6 ± 12.2 years and 84.9% of the patients were male. Serum ALB and T-bil levels were 4.0 ± 0.6 g/dL and 1.13 ± 0.96 mg/dL, respectively, indicating that the majority of patients had mild liver damage. The serum % CDT values and GGT levels were significantly decreased at 73 ± 43 days of abstinence from alcohol when compared with baseline values (*p* < 0.0001; Figure 4A). The levels of Mac-2 bp, AAL-Hp and ALBI were also significantly decreased at 73 days post initiation of abstinence from alcohol (*p* < 0.0001, *p* < 0.001, *p* < 0.001, respectively; Figure 4B). However, *E.*CPS antibody titer level was not changed during the 73-day period of alcohol cessation. Interestingly, *E.*CPS antibody titer values were relatively high in patients with ALD mimicking levels seen in liver cirrhosis patients (Figure 4B). These results suggest that abstinence from alcohol for 1 to 2 months may not be enough to decrease *E.*CPS antibody titer, even though overall liver condition improves.

## Discussion

Our study demonstrates that serum *E.*CPS antibody titer is elevated only in patients with advanced CLD (Child-Pugh B or C), while serum LBP, Mac-2 bp and AAL-Hp levels are elevated not only between investigatory groups (chronic hepatitis and liver cirrhosis) and healthy controls, but between the investigatory groups themselves. Notably, we verified that serum *E.*CPS antibody titer can be used as a prognostic factor in CLD patients independent of serum Mac-2 bp levels or ALBI score. Furthermore, we revealed that serum *E.*CPS antibody titer may indicate the overall condition of the liver-gut axis; rifaximin treatment-induced titer level changes altered OS in liver cirrhosis patients, while titer levels were unchanged by 1–2 months of abstinence from alcohol in ALD patients.

The gut-liver axis affects the progression of CLD and its complications, which leads to enhanced systemic inflammation *via* two routes (2). The first path carries bacterial products from the gut to the liver through portal circulation and eventually systemic circulation. The second path transfers bacteria and other toxins *via* the lymphatic system to the systemic vasculature. The gut-associated lymphoid tissue, including Peyer’s patches and mesenteric lymph nodes, confine the bacteria thus preventing unwanted systemic exposure and subsequent immune response. Previous findings in experimental ALD mice, and in patients with ALD, showed that translocation of intestinal *E. faecalis* to the liver induces ethanol-induced liver inflammation and hepatocyte damage (8). A recent study in patients with cirrhosis showed that intestinal *E. faecalis* increases in parallel with severity of cirrhosis and MELD score (19). However, the correlation between exposure to *E. faecalis* and CLD progression has not been well studied clinically, and no clinical study has investigated the usefulness of serum antibody titer against *E. faecalis* as a predictive marker for survival in cases of CLD. To assess the consequences of *E. faecalis* translocation on immunity, we developed an assay for the detection of serum antibody titer against *E.*CPS according to previous reports (17). We found that serum *E.*CPS antibody titer is elevated only in advanced CLD patients. The elevation of *E.*CPS antibody titer may be associated with the consequences of gut-associated lymphoid tissue inflammation or *E.* bacteremia. Further study is required using a larger cohort, as well as using multiple samples per subject, in order to establish reproducibility.

The ALBI score is a well-known predictive measure for patient prognosis in those who have CLD following liver failure (20). In this study, we showed that CLD patient survival rate can be predicted based on serum *E.*CPS antibody titer greater than 0.1. In addition, serum LBP and Mac-2 bp levels, as well as ALBI score, were also prognostic factors in CLD patients, which is consistent with previous reports (21, 22). We further showed that *E.*CPS antibody titer can be used as an independent predictor of survival, suggesting that *E.*CPS antibody titer may be a more accurate prognostic factor.

A recent investigation has reported that rifaximin significantly decreases the occurrence of overall complications in patients with advanced stages of cirrhosis, leading to prolonged survival (23). Rifaximin may play a role in gut barrier repair, which may be the mechanism by which it ameliorates bacterial translocation and systemic endotoxemia in cirrhosis (24). Changes to intestinal permeability permit gut-derived bacterial products, including *E.*CPS, to infiltrate the portal circulation, lymphoid tissue and systemic circulation. In this study, *E.*CPS antibody titer at baseline, or 3 and 6 months post-rifaximin treatment, were associated with increased OS in liver cirrhosis patients with hepatic encephalopathy. Rifaximin treatment provides potent activity against several species of *E.* bacteremia and a *E.*CPS antibody may be useful in evaluating and monitoring the reactivity to *E. faecalis* product translocation.

There is strong evidence that the gut-liver axis is causatively linked to the progression of ALD and systemic inflammation, both in patients and experimental animal models (25). Indeed, human trials have shown antibiotics and probiotics to be effective in reducing the number of gram-negative bacteria, and altering the gut flora, thus preventing further alcohol-induced liver injury and liver fibrosis (26). Interestingly, we confirmed that the *E.*CPS antibody titer in patients with ALD was relatively higher than the levels observed in liver cirrhosis patients. This evidence may suggest an interaction between the progression of ALD and the consequences of gut-associated lymphoid tissue inflammation or *E.* bacteremia.

Despite our important findings, there are limitations to the present study. This study was retrospective and was conducted within two medical centers using a small number of patients in each cohort. It will be important to examine a multicenter validation study in the future. The effectiveness of rifaximin for prolonging survival, accompanied by an improvement in serum *E.*CPS antibody titer in patients with CLD, should be conducted using a randomized control study. In addition, a comprehensive evaluation of *E.*CPS antibody behavior in ALD patients is also necessary.

In conclusion, the present study illustrated that *E.*CPS antibody titer appears to be a strong predictor of survival in CLD patients. Serum *E.*CPS antibody titer decreases in liver cirrhosis patients receiving rifaximin, and may be associated with an overall improvement in rate of survival.

## Data availability statement

The raw data supporting the conclusions of this article will be made available by the authors, without undue reservation.

## Ethics statement

The studies involving human participants were reviewed and approved by the Mie and Osaka Universities, Mie Prefectural Mental Care Center, and Omiya City Clinic. The patients/participants provided their written informed consent to participate in this study.

## Author contributions

AE and MI contributed to study concept and design, interpretation of data and drafting of the manuscript. YT, RS, RN, HH, JK, MM, and EM contributed to the acquisition of data. EM and HN contributed to study supervision. All authors have read and agreed to the published version of the manuscript.

## Supplementary material

Supplementary Figure 1 Serum biomarker levels in healthy controls, chronic hepatitis and liver cirrhosis groups excluding 18 HCC patients. Changes in LBP, *E.*CPS, Mac-2 bp, AAL-Hp and ALBI. HC, healthy controls; CH, chronic hepatitis; LC, liver cirrhosis; LBP, lipopolysaccharide-binding protein; *E.*CPS, *E. faecalis* capsular polysaccharide; Mac-2 bp, Mac-2 binding protein; AAL-Hp, aleuria aurantia lectin-haptoglobin; ALBI, albumin-bilirubin. **p* < 0.05, ^**^*p* < 0.01, ^***^*p* < 0.001, and ^****^*p* < 0.0001.

Supplementary Figure 2 The contributions of LBP, *E.*CPS, Mac-2 bp, AAL-Hp and ALBI score to overall survival excluding 18 HCC patients. **(A)** Surviving vs. deceased, **(B)** receiver operator characteristic analysis of survival curves, **(C)** survival curves. LBP, lipopolysaccharide-binding protein; *E.*CPS, *E. faecalis* capsular polysaccharide; Mac-2 bp, Mac-2 binding protein; AAL-Hp, Aleuria aurantia lectin-haptoglobin; ALBI, albumin-bilirubin; AUC, area under the curve; CI, confidence interval. **p* < 0.05, ^**^*p* < 0.01.

Supplementary Figure 3 Plots of 1 year C-index for integrated factors with LBP, *E.*CPS, Mac-2 bp and ALBI score. C-index, concordance index; LBP, lipopolysaccharide-binding protein; *E.*CPS, *E. faecalis* capsular polysaccharide; Mac-2 bp, Mac-2 binding protein; ALBI, albumin-bilirubin.
