# Supplementary material for: Elevation of enterococcus-specific antibodies associated with bacterial translocation is predictive of survival rate in chronic liver disease
Source: Front Med (Lausanne). 2022 Aug 11;9:982128. doi: 10.3389/fmed.2022.982128 (PMC9403143; doi:10.3389/fmed.2022.982128)
Supplement: Supplementary file 1 [file Table_1.pdf]

Supplementary Table 1. Demographic and Clinical Characteristics of Subjects excluding HCC patients.

Parameter	CH (N = 59)	LC (N = 32)	P values
Age (yr)	69.0 (58.0, 74.0)	65.0 (54.5, 67.0)	0.0753
Gender (M/F)	31/28	23/9	0.0730
Etiology (HBV/HCV/alcohol)	18/41/0	6/9/17	<0.0001****
Child-Pugh (A/B-C)		17/14	
BMI (kg/m^2)	22.7(19.5, 24.5)	23.3 (20.1, 24.9)	0.5201
AST (U/L)	24.0 (20.0, 31.0)	33.0 (26.3, 43.0)	0.0002***
ALT (U/L)	18.0 (13.0, 25.0)	21.5 (14.3, 28.5)	0.2621
ALB (g/dL)	4.40 (4.20, 4.60)	3.80 (3.40, 4.30)	<0.0001****
T-bil (mg/dL)	0.80 (0.60, 1.00)	1.10 (0.93, 1.60)	<0.0001****
GGT (U/L)	19.0 (15.0, 32.0)	41.5 (22.5, 113.3)	0.0002***
FIB-4 index	2.25 (1.49, 3.28)	4.11 (2.26, 6.80)	0.0001***
Na (mEq/L)	141.0 (140.0, 142.0)	139.0 (137.0, 142.0)	0.0205*
BUN (mg/dL)	14.5 (12.6, 16.6)	14.9 (10.2, 17.0)	0.6597
Cr (mg/dL)	0.73 (0.59, 0.91)	0.80 (0.69, 1.09)	0.0374*

HBV, hepatitis B virus; HCV, hepatitis C virus; HCC, hepatocellular carcinoma; BCLC, balcelona clinic liver cancer; BMI, body mass index; ALB, albumin; ALT, alanine aminotransferase; AST, aspartate aminotransferase; Na, sodium; BUN, blood urea nitrogen; Cr, creatinine; T-bil, total bilirubin; GGT, glutamyl-transferase; FIB-4, Fibrosis-4.

Statistics include number (%) or median (25th and 75th percentiles). **** P < 0.0001, **P<0.01, * P < 0.05.
