# Supplementary figures and images for: Elevation of enterococcus-specific antibodies associated with bacterial translocation is predictive of survival rate in chronic liver disease
Source: Front Med (Lausanne). 2022 Aug 11;9:982128. doi: 10.3389/fmed.2022.982128 (PMC9403143; doi:10.3389/fmed.2022.982128)

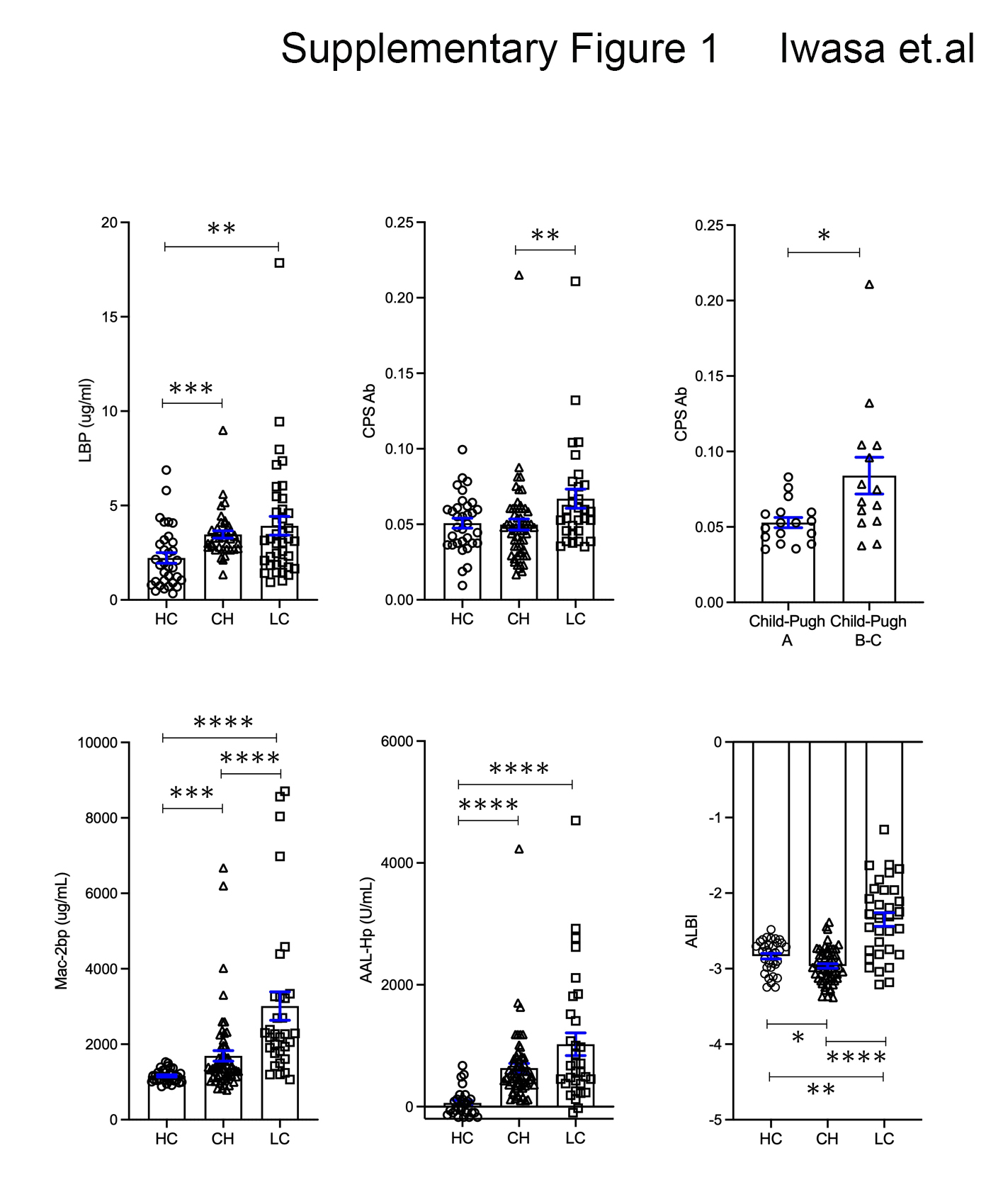

Supplement: Supplementary Figure 1 — Serum biomarker levels in healthy controls, chronic hepatitis and liver cirrhosis groups excluding 18 HCC patients. Changes in LBP, E.CPS, Mac-2 bp, AAL-Hp and ALBI. HC, healthy controls; CH, chronic hepatitis; LC, liver cirrhosis; LBP, lipopolysaccharide-binding protein; E.CPS, E. faecalis capsular polysaccharide; Mac-2 bp, Mac-2 binding protein; AAL-Hp, aleuria aurantia lectin-haptoglobin; ALBI, albumin-bilirubin. *p < 0.05, **p < 0.01, ***p < 0.001, and ****p < 0.0001. [file Image_1.jpg]

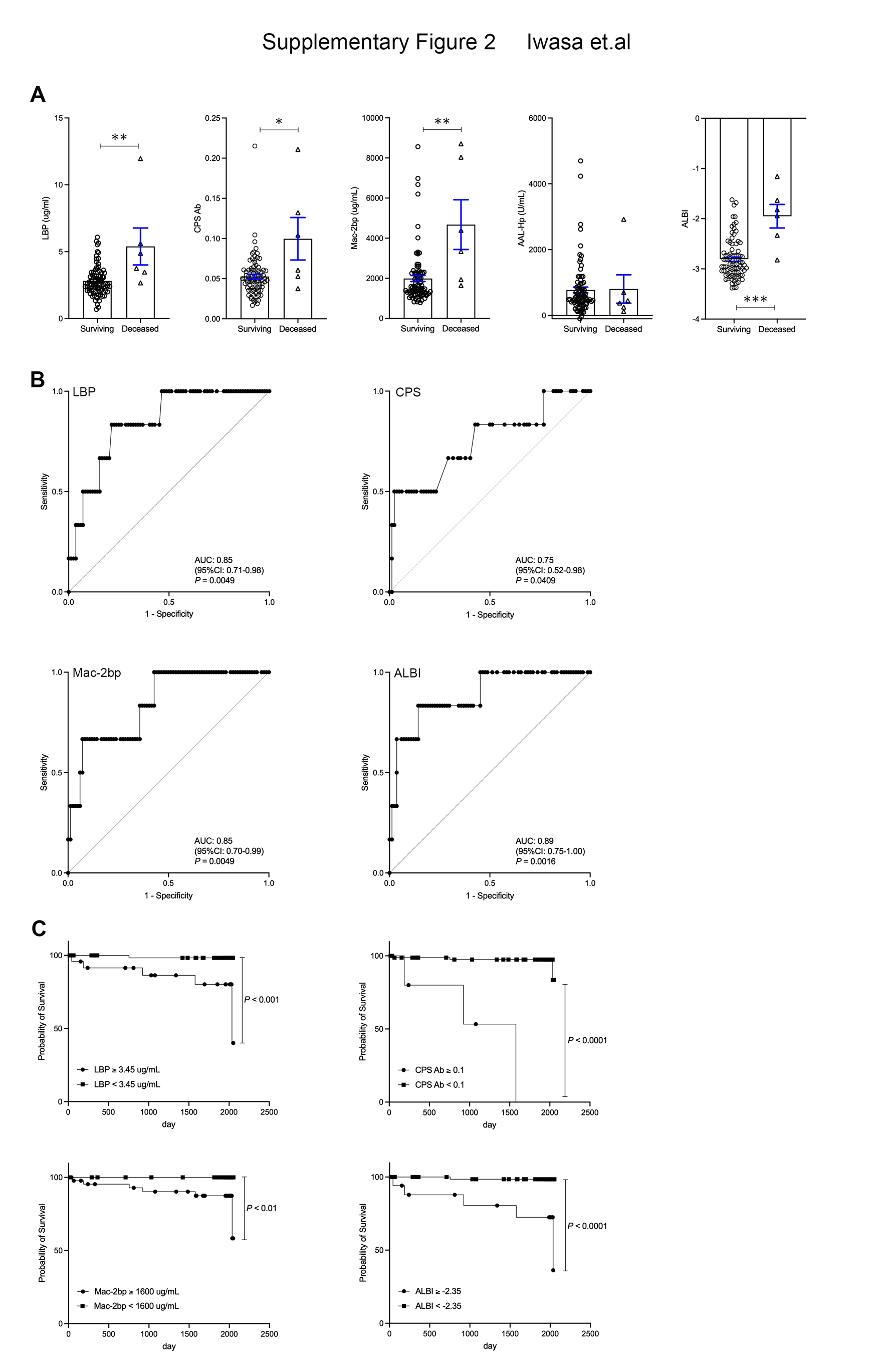

Supplement: Supplementary Figure 2 — The contributions of LBP, E.CPS, Mac-2 bp, AAL-Hp and ALBI score to overall survival excluding 18 HCC patients. (A) Surviving vs. deceased, (B) receiver operator characteristic analysis of survival curves, (C) survival curves. LBP, lipopolysaccharide-binding protein; E.CPS, E. faecalis capsular polysaccharide; Mac-2 bp, Mac-2 binding protein; AAL-Hp, Aleuria aurantia lectin-haptoglobin; ALBI, albumin-bilirubin; AUC, area under the curve; CI, confidence interval. *p < 0.05, **p < 0.01. [file Image_2.JPEG]

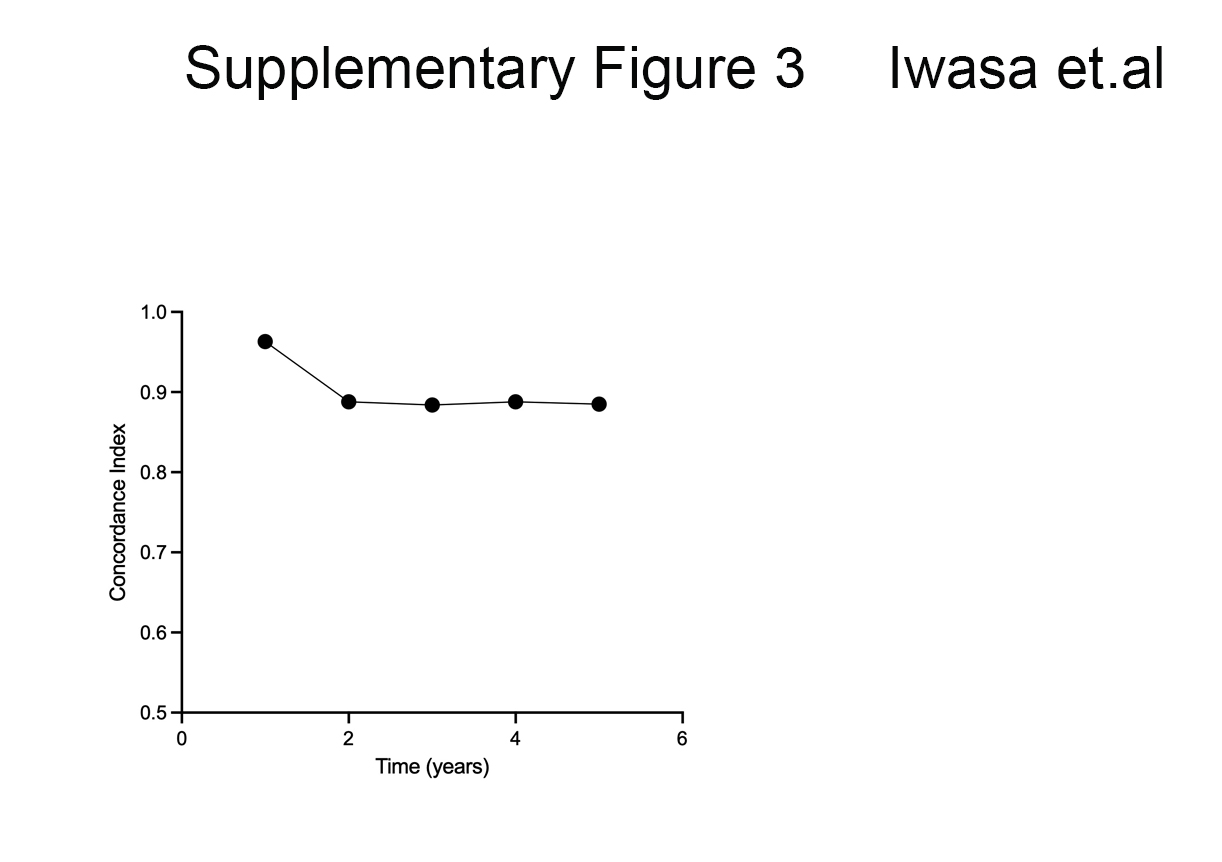

Supplement: Supplementary Figure 3 — Plots of 1 year C-index for integrated factors with LBP, E.CPS, Mac-2 bp and ALBI score. C-index, concordance index; LBP, lipopolysaccharide-binding protein; E.CPS, E. faecalis capsular polysaccharide; Mac-2 bp, Mac-2 binding protein; ALBI, albumin-bilirubin. [file Image_3.jpg]
